# Addressing gaps in AMR awareness in the public: an evidence-based policy brief to guide school curriculum review in Uganda

**DOI:** 10.3389/fpubh.2023.1287523

**Published:** 2023-11-23

**Authors:** JP Waswa, Reuben Kiggundu, Mohan P. Joshi, Joseph Mpagi, Hassan Kasujja, Marion Murungi, Henry Kajumbula, Esther Were, Dan Schwarz, Kamada Lwere, Niranjan Konduri

**Affiliations:** ^1^USAID Medicines, Technologies, and Pharmaceutical Services Program, Management Sciences for Health, Kampala, Uganda; ^2^USAID Medicines, Technologies, and Pharmaceutical Services Program, Management Sciences for Health, Arlington, VA, United States; ^3^Faculty of Health Sciences, Busitema University, Mbale, Uganda; ^4^Department of Microbiology, College of Health Sciences, Makerere University, Kampala, Uganda; ^5^Global Health Systems Innovation, Management Sciences for Health, Medford, MA, United States; ^6^Faculty of Health Sciences, Soroti University, Soroti, Uganda; ^7^Faculty of Health Sciences, Islamic University in Uganda, Mbale, Uganda

**Keywords:** antimicrobial resistance, AMR, curriculum review, public awareness, school education, policy brief

## Abstract

The government of Uganda, through its Ministry of Health, previously adopted curriculum review as a mechanism to respond to public health threats such as HIV/AIDS and include content in primary and secondary schools. This approach contributes to raising public awareness, a key strategy recommended by the World Health Organization to support the global response to the threat of antimicrobial resistance (AMR). This policy brief, developed for policymakers related to school curricula, aims to advocate for and support integration of AMR content in Uganda's primary and secondary level school curricula. The policy brief supports efforts by the multisectoral National AMR Subcommittee to create awareness on this issue as part of its role in facilitating the operationalization of Uganda's National Action Plan on AMR.

## Introduction

Educational curriculum reviews are an innovative and effective approach for responding to public health threats by, for example, including topics related to TB and HIV/AIDS (1–6). Prior to 2004, HIV/AIDS education did not constitute a formal component of the Ugandan school curriculum; it was imparted mainly through alternative extracurricular channels such as media, youth groups, drama, music, and parent-teacher associations. The impetus for a more formal inclusion of HIV/AIDS information in the national curriculum came from the perspectives of schools and stakeholders (7). This approach fostered public education and awareness about HIV/AIDS, and lessons learned through the many years of its implementation led to the identification of sexuality education for young people as an additional mechanism for strengthening knowledge about HIV/AIDS (8). This process and its outcome demonstrate the effectiveness of responding to public health threats through formal instruction in the national curriculum and additional teaching mechanisms.

The World Health Organization (WHO) Global Action Plan on antimicrobial resistance (AMR) recommends that countries include AMR and antimicrobial-use topics in school curricula in order to promote better understanding and awareness of the issue and provide the public with accurate and relevant information (9). WHO has developed guidance on AMR education and training for health workers but not for pre-tertiary education (10). Education on AMR has a significant influence on antimicrobial consumption and should be implemented while considering other social and economic factors that influence AMR (11, 12). Evidently, education has a central role to play in combating the surge of AMR, and some countries, such as the United Kingdom, have adopted education strategies dedicated to AMR that cover both health care and community education (including school students, society and non-health care professional students) (12, 13).

In this policy brief, we recommend considering the inclusion of at least some basic AMR-related content for all levels of school education in Uganda, specifically primary and secondary levels. We provide context regarding the burden of AMR in Uganda and its linkage to the wider global burden of drug-resistant infections and efforts to combat the problem. We then provide an AMR-related policy context for Uganda, specifically focusing on WHO's Global Action Plan and Uganda's National Action Plan on AMR (NAP-AMR) and National Action Plan for Health Security (9, 14, 15). After outlining the current efforts to contain AMR, we present an analysis of current primary and secondary school curricula and gaps identified with respect to AMR training. Finally, we make recommendations aimed primarily at policymakers for considering curriculum reviews to address these gaps in support of comprehensive efforts to combat AMR in Uganda.

## The burden of AMR in Uganda

Like many countries with a high burden of infectious diseases, Uganda relies heavily on antimicrobials to treat those diseases (16). That, in conjunction with the country's limited resources, has made the challenge of combating AMR a priority concern for Uganda. A significant proportion of bacteria in Uganda have exhibited high resistance rates—often up to 50%—against commonly prescribed antibiotics such as penicillin, cephalosporins, tetracyclines, and trimethoprim-sulfamethoxazole (17). The prevalence of multi-drug resistant bacteria, including methicillin-resistant *Staphylococcus aureus* (MRSA) and those that produce extended-spectrum beta-lactamase, is on the rise, and these pathogens are displaying increasing resistance to even antibiotics that are generally reserved for tough multi-drug resistant infections (17–19).

Although some AMR could develop naturally, its rapidly increasing prevalence is driven mainly by the overuse and misuse of antimicrobials, particularly antibiotics (20). Inappropriate antibiotic use in communities results from unregulated over-the-counter access and use, inadequate health care infrastructure, limited awareness among the general public, and non-biomedical factors such as self-medication and storing antibiotics at home (21, 22). Health system challenges are a key factor in driving the documented inappropriate antibiotic use in health care facilities (23, 24). Furthermore, poor hygiene and sanitation practices, including inadequate access to water, sanitation, and hygiene facilities, and inadequate infection prevention and control practices in health facilities encourage the transmission of resistant pathogens (25, 26). As such, the most significant strategies for combating AMR aim to eliminate the unnecessary use of antimicrobials in humans and animals and prevent the transmission of infectious pathogens in health facilities and communities.

One of the strategic objectives of Uganda's NAP-AMR aims to improve knowledge and awareness of AMR among health practitioners, farmers, and the general public (14). A significant level of knowledge about AMR has been documented among health care providers and clinical students in Uganda (27, 28). Inadequate public awareness and knowledge about AMR has been documented in low- and middle-income countries in sub-Saharan Africa, resulting in antibiotic misuse by the general public (29–31). While such documentation regarding public awareness and knowledge is not available specifically for Uganda, the situation is likely to be similar there based on documented antibiotic misuse by the general public (32, 33). Uganda's public continues to receive low levels of exposure to information about AMR and its drivers, as is generally the case in most countries (34).

## AMR content-related gaps identified in primary and secondary school curriculum in Uganda

In pursuit of its goals and aspirations, Uganda, like other countries, uses its education system as a vital tool. To ensure the provision of high-quality education, Uganda is implementing comprehensive curricula tailored to various education levels, framework consisting of 7 years of primary education, followed by 6 years of secondary (senior) education comprising 4 years of lower secondary also known as ordinary level (O-level) and 2 years of upper secondary school also known as advanced level (A-level); and finally, 3 to 5 years of post-secondary education (35).

The curricula are developed by the National Curriculum Development Center (NCDC) of the Ministry of Education and Sports (MOES). All schools are expected to follow the same curricula, with any additional external curricula requiring approval from the National Curriculum Development Center and MOES before implementation (35).

### Methods

Analysis of the Uganda education and training curricula for AMR and related content, using a set of keywords, was conducted in August 2022 to understand the scope and depth of the content covered and its relevance to AMR containment efforts. The exercise, coordinated by Makerere University Biomedical Research Center, involved key stakeholders, including the National Curriculum Development Center, MOES, and health professional councils. This keyword analysis technique was employed to examine and analyze textual data, allowing for the identification and exploration of specific keywords and key phrases within curricula documents to gain insights, detect patterns, or extract relevant information related to AMR content (36). The curricula-related documents analyzed included syllabi for primary and secondary levels of education. Portable document format versions of primary and secondary syllabi covering all currently taught subjects were acquired. They were systematically searched for specific keywords and key phrases related to topical areas of resistance to antimicrobials, infection prevention and control, and antimicrobial use. The keywords and key phrases were grouped into 27 analytical “terms”—four related to resistance to antimicrobials, 12 related to infection prevention and control, and 11 related to antimicrobial use (Box 1). This method allowed for a focused exploration of the syllabi, enabling the identification and analysis of the sentences and paragraphs where the keywords and key phrases were mentioned and relating them to AMR. Once this relationship was established, the scenario was recorded as a “hit.” For all the syllabi, the search terms with hits were noted and AMR content was defined as a proportion of “terms with hits” among the 27 search terms analyzed.

Box 1 **Search terms (keywords and key phrases) used for analysis of school curricula of Uganda.**

***Resistance to antimicrobials***

1. Microorganisms/germs/bacteria
2. Antimicrobial/antibiotic resistance
3. Sensitivity
4. Pesticides/insecticide

***Infection Prevention and Control***

1. Infection/infectious diseases
2. Water borne diseases/zoonoses
3. Sanitation
4. Hygiene
5. Handwashing/hand hygiene
6. Soap
7. Alcohol-based hand rub/sanitizer
8. Cough
9. Rubbish/waste
10. Waste disposal/segregation
11. Immunization/vaccination
12. Toilet/latrine

***Antimicrobial use***

1. Drugs/medicines/antibiotics
2. Treatment
3. Appropriate use of medicines/prescription/prescribe
4. Antimicrobial
5. Drug resistance
6. Dose/dosage
7. Adherence/adhere
8. Administer/give
9. Drugs storage
10. Drug manufacture
11. Drug records/information/record keeping

### Results

**Figure 1:**
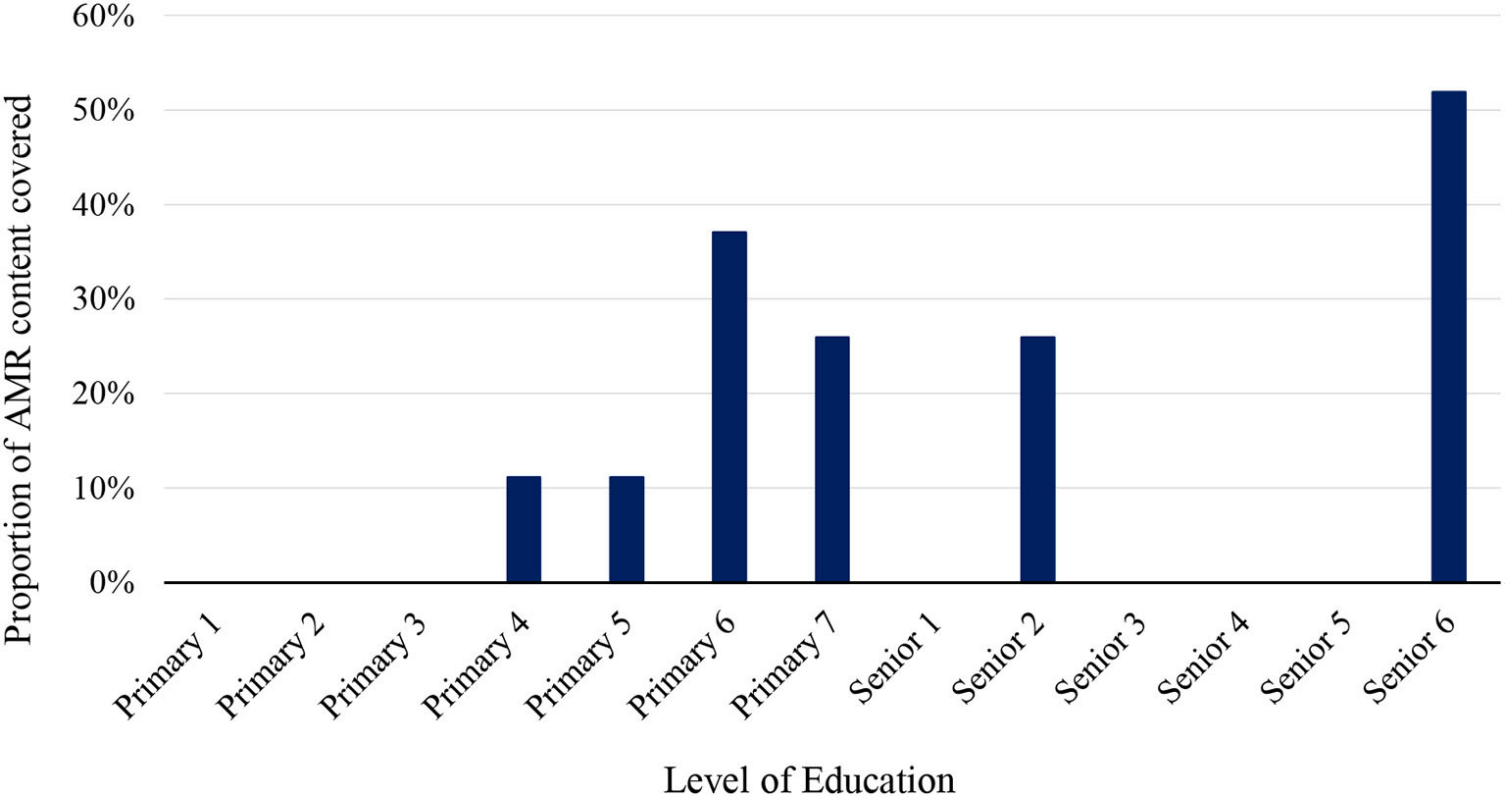
AMR content in the curricula at various levels of primary and secondary (senior) school education in Uganda. Official learners' ages: primary 1 to 7 (5–12 years), senior 1 to 4 (13–16 years) and senior 5 to 6 (17–18 years) (37).

Figure 1 shows the proportion of AMR content as “terms with hits” at the various levels of primary and secondary education curricula analyzed. Content on AMR was first mentioned at the primary 4 level, with cumulatively more content thereafter. AMR content is absent in the majority of secondary school levels. Only senior 3 and senior 6 had content on AMR with the latter, the highest and last level of secondary level education, containing above 50% of AMR content. This shows that there are opportunities to increase exposure to AMR content in a more continual and comprehensive way throughout primary and secondary levels.

In 2006, the Government of Uganda started implementing a strategic policy mandating science education for all learners in primary and lower secondary levels of education (38). Science subjects are optional for learners in the upper secondary level of education. It is significant to note that in the upper secondary level of education, AMR content is only included for students undertaking science, technology, engineering, and mathematics (STEM)-based subjects. In 2022, only 39.6% (38,765/97,890) of students opted for at least one STEM subject (39), with the rest opting for the humanities and thus having no exposure to AMR content. This creates a further significant opportunity to increase exposure to AMR content outside the STEM curriculum.

## Policy options and implications

Uganda progressively built capacities for AMR containment over the last few years (40–42). Uganda ratified the NAP-AMR for 2018–2023 in 2018 (14) and is currently making various efforts to operationalize it. The country's AMR containment efforts have also been guided by the recommendations contained in various WHO documents, such as the Global Action Plan on AMR (9), benchmarks for International Health Regulations (IHR) capacities (43), and the second edition of the Joint External Evaluation tool (44). In doing so, the country adopted a One Health approach that uses a multisectoral coordination strategy. Despite some progress having been made, gaps still exist in implementing Strategic Objective 1 of the NAP-AMR, which relates to public awareness, training, and education.

WHO developed a curriculum framework for health workers to guide AMR curriculum inclusion for appropriate knowledge, skills, and attitude (10). However, achieving the NAP strategic objective on training and education will require creation of a whole of society education plan, not only focusing on health workers but also implementing relevant pre-tertiary and pre-service curriculum reforms to create wider awareness of the burden of AMR. The MOES has an opportunity to increase exposure to AMR content in a more continual (the timing and frequency of content) and comprehensive (the amount of content) manner throughout public education as a mandated core curricular goal to improve and protect the public health of Uganda for the future.

AMR clearly illustrates the interdependence of human, animal, and environmental health, with the drivers and impacts of AMR experienced across all three sectors. The Government of Uganda made some efforts to implement multi-sectoral approaches for combating AMR, including by adopting the One Health approach and implementing the NAP-AMR. The policy brief presents a policy option with an additional opportunity for the line ministries working in One Health and MOES to draw lessons from NAP-AMR implementation and expand the scope of AMR content in school curricula to introduce the One Health concept at the primary level and continuously expand on those lessons in the secondary levels.

Implementing this policy has potential implications for the AMR response. Introduction of AMR content early in the education system could strengthen the One Health response at all levels through creating knowledge about the AMR burden as a One Health challenge. The increased public awareness will ensure that the public takes more responsibility in the use of antibiotics and prevention of infections. Secondly, farmers, who constitute over 80% of the national workforce will be better equipped on handling antibiotics and other One Health related issues, contributing to better implementation of One Health actions through the knowledge gained through their earlier years in school. There is potential impact on the future designing and implementation of AMR containment activities in Uganda, with the potential of this policy brief to inform the planned revision of the Uganda NAP scheduled for 2024.

## Actionable recommendations

Incorporating AMR training into existing education curricula can be a low-cost and sustainable strategy for countries to address AMR. This should be done as early as possible, with specific objectives in mind, to educate not only future health care workers but also the public about AMR. One effective approach could be to integrate AMR content into the school education curricula and extracurricular activities starting with pre-primary and primary levels and then continuing in secondary and tertiary levels of education. Targeting to introduce young children to some very simple concepts about AMR and its containment early could be a strategically strong approach for Uganda due to the high rates of primary school completion in the country (37). The content could then gradually increase in complexity and quantity as learners progress through the years. A similar approach has been successfully utilized in Uganda to incorporate HIV awareness into the education system, and the same principle can be applied to AMR-related education (45). Moreover, enhancing and broadening the current curriculum by revising and expanding existing content to encompass a wider scope of AMR, One Health, and global health security concepts is crucial. For instance, while educating students about the importance of hygiene and hand washing in preventing AMR, opportunities exist to introduce and connect with other comprehensive concepts, such as disease outbreaks and pandemics. In early primary education, the curricular exposure should be comprehensive enough to expose all learners to AMR content, and while some learners may later opt-in to more complex science curricula that digs deeper into technical details while others choose art-based curricula, that should not prevent curriculum developers from finding ways to incorporate AMR content into non-science curricula, i.e., incorporate public health messages into art classes.

Inclusion of AMR education into various regular school activities provides a significant opportunity for early AMR training. For primary school students, AMR-related concepts can be introduced through plays and fun-filled activities—including educational bingo, music and singing, and outdoor learning activities—to facilitate knowledge acquisition and retention, cognitive performance, and healthy development (46, 47). And as children rise in education levels, school debates, interest clubs and other interactive and extracurricular activities can be introduced to allow students to engage in discussions and learn about AMR in more practical ways (48, 49). Incorporating extracurricular activities related to AMR into the standard school curriculum can be an effective strategy for early education and training on AMR (50). One study found that a debate lesson significantly improved students' knowledge on antibiotic use for treatment of colds and its effect on development of AMR (51). Furthermore, themes related to AMR could be included in competitive school activities, such as national competitions for music, drama, and science, thereby encouraging learners to creatively explore and raise awareness about the topic. A primary school musical about AMR improved both short-term and long-term knowledge about AMR among 9 to 11-year-old children in England, demonstrating the effectiveness of musical theater as an educational tool for fostering education and training on AMR (52).

To further underscore the significance of AMR containment, it is crucial to incorporate questions related to AMR in national examinations and school progressive assessments. By including such questions, learners will be motivated to delve into the subject matter and enhance their understanding and knowledge of AMR. Examinations have been shown to facilitate knowledge acquisition, especially as a result of exam preparations, and to improve memory and modulate memory formation (53). This integration ensures that AMR becomes an integral and essential component of children's education, fostering a greater awareness of the issue among the student population and, as a potential spill-over effect, among their parents and other family members.

To promote behavior-change at the community level, undertaking community-based education initiatives aimed at empowering local communities to understand AMR and their role in the fight against it can be of great significance. These initiatives reach a relatively high number of people in the communities and have been demonstrated to have a wide impact on antimicrobial use at population level (54). The initiatives can include educational sessions (e.g., focus group discussions), workshops, and outreach programs conducted at the grassroots level, targeting diverse stakeholders including village/community health workers, farmers, and community leaders. Through interactive sessions and accessible materials, the community learns about responsible antibiotic use, the impact of AMR on health and agriculture, and the importance of the One Health perspective. By fostering awareness and promoting behavioral changes, community-based education plays a vital role in curbing AMR and promoting sustainable health practices.

It is recommended that sections dedicated to the theme of AMR be created in the national museum as well as school and public libraries. Such an AMR section would serve as an educational hub, offering a comprehensive showcase of information on AMR. Visitors would have the opportunity to explore the history of antimicrobials, understanding their development and impact on health care. The section would provide valuable insights into the progressive rise of AMR, illustrating the factors contributing to its emergence and spread and the challenges it poses to global health. Exhibits and literature would highlight the consequences of AMR, such as the case of chloroquine—a once-golden drug for treating malaria that became obsolete due to unacceptable levels of resistance, which in turn led to the revision of national policies on malaria (55). This visual representation would emphasize the urgent need for the responsible use of antimicrobials. Furthermore, such a section would elucidate the implications of AMR on health care provision, offering a broader perspective on the potential consequences of unchecked AMR. By presenting real-world examples and case studies, visitors would gain a deeper understanding of the negative impact of AMR on treatment, patient survival, public health, medical advancements, and cost to individuals and health systems. In addition to static exhibits, interactive displays, multimedia presentations, and engaging demonstrations could be incorporated to enhance the visitor experience. This would encourage active learning and provide opportunities for hands-on exploration of AMR-related concepts. Ultimately, the establishment of a dedicated AMR section in museums and libraries would serve not only as a valuable resource for education and awareness but also as a platform for fostering collaboration and dialogue among scientists, health care professionals, policymakers, and the public. Through this immersive experience, visitors would gain a heightened appreciation for the importance of, and responsibilities for, addressing AMR as a critical global health concern, including as a health security threat.

## Conclusion

AMR is a pressing global health issue that demands urgent attention. The low public awareness about AMR is among several significant risks to AMR containment efforts. Curriculum review to introduce content on AMR through early learning at primary and secondary levels of school education presents an easy, low-cost, innovative, and sustainable opportunity to help raise widespread awareness on AMR. Since completion of a primary level education is nearly universal in Uganda and the completion rate for secondary schooling is over 70%, the country can make significant strides toward its goal of AMR containment through the integration of AMR contents in school curricula. This can be a major intervention in support of public awareness, training, and education, which is currently neither systematically addressed nor well-funded even though it is a key pillar of Uganda's NAP-AMR.

## Author contributions

RK and JPW conceived the idea. RK, JPW, and MPJ wrote the first draft. JPW, RK, MPJ, JM, HKas, MM, HKaj, DS, KL, and NK each contributed knowledge and ideas. JPW, RK, MPJ, DS, and NK contributed to critical revisions. JPW, MPJ, and NK finalized the paper. All authors reviewed and approved the final version.
